# Enrollment in Treatment at a Specialized Pain Management Clinic at a Tertiary Referral Center after Surgery for Ulnar Nerve Compression: Patient Characteristics and Outcome

**DOI:** 10.1016/j.jhsg.2021.02.001

**Published:** 2021-03-08

**Authors:** Alice Giöstad, Ronja Räntfors, Torbjörn Nyman, Erika Nyman

**Affiliations:** ∗ Department of Biomedical and Clinical Sciences, Linköping University, Linköping, Sweden; † Department of Medical and Health Sciences, Pain and Rehabilitation Center, Linköping University Hospital, Linköping, Sweden; ‡ Department of Hand Surgery, Plastic Surgery and Burns, Linköping University Hospital, Linköping, Sweden

**Keywords:** Pain, Patient-reported outcome measures, Ulnar nerve compression syndromes

## Abstract

**Purpose:**

To study patients who enroll in treatment at a specialized pain management clinic at a tertiary referral center following ulnar nerve decompression.

**Methods:**

Data from medical charts and postoperative questionnaires were collected for all patients after surgery for ulnar nerve compression at the elbow from 2011 to 2014 (n = 173) at a tertiary referral center. Differences in characteristics between patients who enrolled in treatment at the pain management clinic (study group, n = 26) and the rest of the patients (reference group, n = 147) were analyzed. The study group was further evaluated using questionnaires from the Swedish Quality Registry for Pain Rehabilitation (SQRP) and regarding outcome of pain treatment.

**Results:**

The study group was characterized by prior pain conditions, earlier contact with a pain management clinic, and high degrees of kinesiophobia, depression/anxiety, low quality of life, and low life satisfaction. These patients had significantly higher postoperative Disabilities of the Arm, Shoulder, and Hand (DASH) scores, were significantly younger, and had bilateral surgery significantly more often than the reference group. For patients with unilateral surgery, simple decompression was significantly more common in the reference group. The most common treatments at the clinic were antidepressants and anticonvulsants for neurogenic pain. In 5 of 26 patients, pain relief, or pain reduction was the documented reason for discharge.

**Conclusions:**

Pain is a relevant outcome measure for ulnar nerve decompression among complicated cases at a referral center. Severe postoperative pain is connected to higher disability, reduced life satisfaction, and overall low health status. This study maps out characteristics of patients who postoperatively enroll in treatment at a specialized pain management clinic following ulnar nerve decompression. Further studies are needed to define predictive factors for such pain.

**Type of study/level of evidence:**

Prognostic III.

Ulnar nerve compression at the elbow is a common cause of peripheral neuropathy in the upper extremity, and it has been studied thoroughly regarding the postoperative outcome. The outcome often focuses on objective measures, and disability is evaluated using validated questionnaires.^1^^,^^2^ Postoperative pain is rarely highlighted or even reported as a complication or a persistent symptom after surgery.^3^ Extensive exclusion criteria are frequently applied in studies comprising a large portion of a whole population, such as patients with revision surgery or patients with comorbidity, like ipsilateral neuropathies.^4^, ^5^, ^6^, ^7^ Revision surgery may also indicate poor results.^7^^,^^8^ Surgery sometimes fails to relieve preoperative pain;^9^ thus, evaluating pain as an outcome after surgery should not be neglected.

Depression, anxiety, and psychological vulnerability before surgery are associated with poor results, increased disability, greater pain intensity, and a higher risk of developing chronic or persistent postsurgical pain in the chest, head, neck, extremities, and abdomen.^10^, ^11^, ^12^ Concurrent or past pain, female sex, and young age are predictors for increased postoperative pain.^13^ Pain in general, and neurogenic pain in particular, affects the quality of life, sleep duration/quality, and psychological well-being.^14^

The presence of neurogenic pain, ie, pain caused by a lesion or disease of the somatosensory nervous system, including peripheral fibers, is often associated with sensory and motor deficits.^15^ Neurogenic pain needs a different approach from nociceptive pain as common pharmaceuticals, including opioids, have little or no effect. Both pharmacological treatment options and their effectiveness against neurogenic pain are limited; 40% to 60% experience partial pain relief.^16^

Surgeons tend to overprescribe opioids after upper extremity surgery.^17^ In a recent study, 14% of patients with ulnar nerve decompression at the elbow were considered to have prolonged opioid use, defined as prescription continued 90 days after surgery.^18^ Notably, opioids may not be necessary for such a procedure. After elective soft tissue hand surgery, including carpal tunnel release, non-opioid drugs have been shown to be as effective as opioids in controlling pain.^19^^,^^20^

In our system, enrollment in treatment at a specialized pain management clinic indicates that surgery failed to relieve the preoperative pain or resulted in severe pain. Pain is defined, by the International Association for the Study of Pain, as an unpleasant sensory and emotional experience associated with, or resembling that associated with, actual or potential tissue damage. Pain is a subjective experience that varies among patients and is influenced by biopsychosocial factors.^21^ We chose enrollment in treatment at a pain management clinic as our primary outcome as it reflected the severity and complexity of the situation and the need for specialized care, regardless of objective measures. Our aim was to investigate patients who enrolled in treatment at a specialized pain management clinic at a tertiary referral center, as a primary outcome measure following surgery for ulnar nerve compression at the elbow, and to study patient characteristics and outcome of treatment at such a clinic. We hypothesized that this population experienced depression, anxiety, and fear of movement, as well as low life satisfaction.

## Materials and Methods

A retrospective study included patients postoperatively enrolled in treatment at the Pain and Rehabilitation Center, Linköping University Hospital, Linköping, Sweden, from January 1, 2011, to December 31, 2014, following surgery for ulnar nerve compression at the elbow at the Department of Hand Surgery, Plastic Surgery, and Burns, Linköping University Hospital, Linköping, Sweden, a tertiary referral center. The surgical techniques employed were simple decompression or subcutaneous transposition as index surgery (n = 150) and subcutaneous transposition (n = 31) as revision surgery after simple decompression. Patients who postoperatively enrolled in treatment at the pain management clinic due to pain arising or worsening after the surgery were the study group, while the reference group referred to the rest of the patients having surgery for ulnar nerve compression at the same referral center during the same period. Patients already preoperatively enrolled at the pain management clinic due to ulnar nerve condition or another pain condition were excluded, as well as those postoperatively enrolled due to other pain conditions (n = 8). These patients were found in the reference group, as this study focused on postoperative pain after surgery for ulnar nerve compression at the elbow.

Data from medical charts (Table 1) and a postsurgical survey were collected, including the Disabilities of the Arm, Shoulder, and Hand (DASH) questionnaire sent out during April 2016 (follow-up time between 16 and 64 months) to all patients, along with information about the study and a written consent form.^9^^,^^22^ Our center did not routinely collect patient-reported outcomes and, as such, preoperative DASH scores were not available. Comorbidities were identified from medical charts and International Classification of Diseases codes. Four different questionnaires from the Swedish Quality Registry for Pain Rehabilitation form (SQRP) were also completed by the patients in the study group. Each patient was assigned a study number, and medical charts and questionnaires were coded. Ethical approval was obtained from the Regional Ethics Review Board, Linköping, Sweden (register number 2016/88-31).

**Table 1:** Characteristics and Comorbidities in Study Group and Reference Group

	Study Group (n = 26)		Reference Group (n = 147)		*P* Value
	Mean (SD)	n (%)	Mean (SD)	n (%)	
**Characteristics**					
Age	42 (10)		51 (14)		**<.001**∗
Female gender		20 (77)		85 (58)	.07‡
BMI	29 (5)		27 (5)		.09∗
Smoking		11 (42)		43 (30)	.19‡
Preoperative McGowan grade					
McGowan grade 1		11 (42)		36 (25)	.14‡
McGowan grade 2		6 (23)		54 (37)	
McGowan grade 3		9 (35)		57 (38)	
Interpreting needs		2 (8)		1 (1)	.06†
Marital status					
Single		4/24 (17)		21/123 (17)	1.00†
Partner		20/24 (83)		102/123 (83)	
**Comorbidity**					
**Type of surgery**					
Unilateral					
Simple decompression		6 (23)		82 (56)	**.002**‡
pAST		5 (19)		28 (19)	1.00†
sAST		7 (27)		16 (11)	.053†
Bilateral		8 (31)		21(14)	**.048**†
Neuropathy in the ipsilateral arm		17 (65)		76 (52)	.20‡
Neuropathy in the contralateral arm		10 (39)		51 (35)	.71‡
Shoulder problems^a^		7 (27)		22 (15)	.16†
Neck problems^b^		9 (35)		31 (21)	.13‡
Depression		5 (19)		13 (9)	.16†
Diabetes		2 (8)		19 (13)	.74†
Cardiovascular disease		6 (23)		43 (29)	.52‡

∗ t test.

‡ χ^2^ test.

† Fisher’s Exact Test.

a Different conditions (such as osteoarthritis, shoulder impingement syndrome, etc.) diagnosed in the medical charts.

b Different conditions (such as disk herniation, whiplash, etc.) diagnosed in the medical charts. pAST, primary anterior subcutaneous transposition. sAST, secondary anterior subcutaneous transposition.

The SQRP was a national registry based on several surveys postoperatively completed by the patient in conjunction with the first visit to a pain management clinic. Hence, no data of this kind or preoperative data were available for the reference group.^23^ The questionnaires from SQRP analyzed in this study were: (1) Tampa Scale of Kinesiophobia (TSK), (2) Hospital Anxiety and Depression Scale (HADS), (3) health status (EQ-5D-3L), and (4) life satisfaction (LiSat-11).

TSK inquires about the patient’s fear of movement-related to pain, and it was used extensively both in trials and clinical services.^24^ The Swedish version was tested on patients with lower back pain and was considered a relevant and reliable questionnaire with both face and content validity.^25^ It comprised 17 items, with each item scored 1 to 4 on a Likert Scale (1 = “strongly disagree” and 4 = “strongly agree”). After the inversion of items 4, 8, 12, and 16, a total score was calculated, ranging from 17 to 68, with a high value indicating a high degree of kinesiophobia.^25^ A score of >37 was set as the cut-off for indicating kinesiophobia.^26^^,^^27^

HADS was a widely used valid scale for measuring anxiety and depression in somatic hospital care.^28^ It consisted of 2 parts, HADS Anxiety and HADS Depression. For each of these subscales, the score varied from 0 to 21. A cut-off value of ≥8 was considered to predict both depression and anxiety.^29^

EQ-5D-3L consisted of 5 dimensions regarding health-related well-being with 3 different severity levels and a visual analog scale (VAS) for current health status (ranging from 0 to 100, with 0 representing worst possible health and 100 best possible health). The answers from the 5 questions were summarized as an index number from −1 to 1, where −1 was very low health-related well-being and 1 was excellent health-related well-being.^30^

LiSat-11 measured life satisfaction through 11 different questions about social life, family, relationships, physical and psychological health, economy, and life in general. Each item was scored 1 to 6, where 1 was very unsatisfied and 6 was very satisfied.^31^^,^^32^

The study group was compared to the reference group regarding patient characteristics, preoperative McGowan grade, comorbidity, surgical method, and postoperative DASH scores. Chi-square test was used when analyzing dichotomized qualitative data; if assumptions were violated, Fisher exact test was applied. The t test was used for continuous normally-distributed data. If conditions were violated, a nonparametric test was used. Parametric data were presented as mean ± standard deviation and nonparametric as median (interquartile range 25^th^-75^th^ percentiles).

## Results

Twenty-six (15%) of 173 patients surgically treated for ulnar nerve compression at the elbow were subsequently enrolled in treatment at the pain management clinic due to severe postoperative pain. Of these, 18 patients had previously been treated for a pain condition at some medical institution before surgery. Ten of these 18 patients had been treated at a pain management clinic but had since been discharged**,** most due to a pain condition unrelated to ulnar nerve compression. No such data for the reference group were available for comparison. Patient characteristics and differences between the study group (n = 26) and the reference group (n = 147) are presented in Table 1. The most common complication after surgery for the study group was postoperatively-emerging neuropathic pain (reported for 12 patients). Other complications were loss of nerve function (4 patients) and complex regional pain syndrome (4 patients). Loss of nerve function incorporated partial or complete loss of both sensory functions, including hypoesthesia and allodynia around the operated area as well as motor function. No postoperative hematomas or infections were reported.

**Figure 1:**
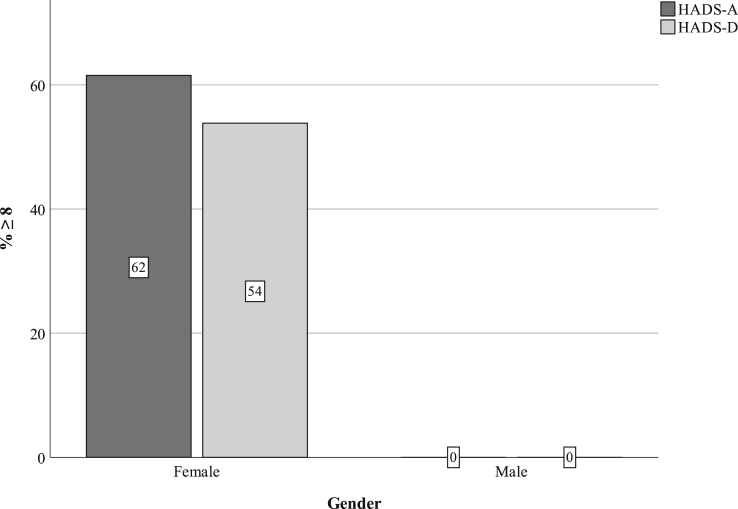
The percentage of HADS scores ≥8 among patients enrolled to a specialized pain management clinic following ulnar nerve compression at the elbow divided by gender is shown. HADS-A, Hospital Anxiety and Depression Scale–Anxiety subscale. HADS-D, Hospital Anxiety and Depression Scale–Depression subscale.

In the SQRP surveys for the study group, scores from the TSK were calculated for 9 patients and found to be 40.3 ± 9.9. Six of 9 patients had a high degree of kinesiophobia (cut-off at 37 points). Fifteen HADS scores were calculated for each subscale (Table 2 and Fig. 1). Patients who scored less than 8 points had a lower mean age than patients with a score of 8 or above (36 years [range, 23–43 years] and 47 years [range, 37–52 years], respectively, *P* =.037, Mann-Whitney U test).

**Table 2:** EQ-5D-3L Frequencies From the Study Group Reported by Dimension and Level

	No Problems; n (%) (Level 1)	Moderate Problems; n (%) (Level 2)	Severe Problems; n (%) (Level 3)
Mobility	6 (55)	5 (45)	0
Self-care	7 (64)	4 (36)	0
Usual activities	2 (20)	6 (60)	2 (20)
Pain/discomfort	0	4 (36)	7 (64)
Anxiety/depression	3 (27)	4 (36)	4 (36)

Responses from the EQ-5D-3L questionnaire are divided by dimension and severity level. Decimals are avoided, given that the rows total 100 ± 1%.

**Figure 2:**
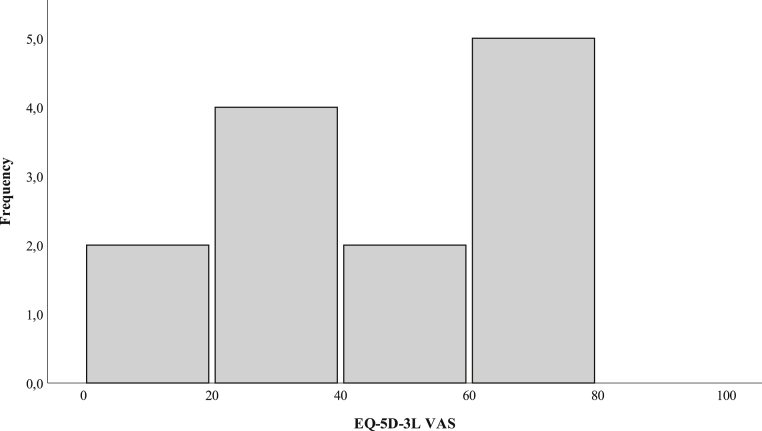
The frequency distribution of EQ-5D-3L VAS among patients enrolled at a specialized pain management clinic at a tertiary referral hospital following ulnar nerve compression at the elbow is shown. Patients’ own assessment of their health on VAS varies from 0 to 100, where 100 represents the best possible health and 0 the worst possible health.

EQ-5D-3L index and VAS current health status values were obtained for 13 patients. EQ-5D-3L index value and mean EQ-5D-VAS were 0.25 (SD 0.32) and 42.5 ± 22, respectively (Fig. 2 and Table 3). Answers obtained for each domain in the LiSat-11 questionnaire varied between 10 and 11. The frequency of the dichotomized answers for each domain in the LiSat-11 questionnaire can be seen in Table 4. No patient scored “very satisfied” or “satisfied” in the domain of physical health (data not shown).

**Table 3:** Study Group Responses to LiSat-11 Dichotomized Into Gross Levels

	Satisfied	Dissatisfied
Life as a whole	50%	50%
Vocation	20%	80%
Economy	27%	73%
Leisure	9%	91%
Contact with friends	18%	82%
Sexual life	40%	60%
Self-care	40%	60%
Family life	55%	46%
Relationships	63%	38%
Physical health	0%	100%
Psychological health	36%	64%

Decimals are avoided, given that the rows total 100 ± 1%.

**Table 4:** HADS Responses by Patients Enrolled at a Specialized Pain Management Clinic at a Tertiary Referral Hospital Surgically Treated for Ulnar Nerve Compression at the Elbow

	Mean (SD)	Median (IQR)	Score ≥8% (n = 15)
HADS-A	9.5 (6.9)	8 (3–16)	53% (8/15)
HADS-D	7.7 (5.2)	7 (3–13)	47% (7/15)

HADS-A, Hospital Anxiety and Depression Scale–Anxiety subscale*.* HADS-D, Hospital Anxiety and Depression Scale–Depression subscale*.* A value of 8 or higher indicates possible or actual depression or anxiety disorder for both subscales.^28^^,^^29^

**Figure 3:**
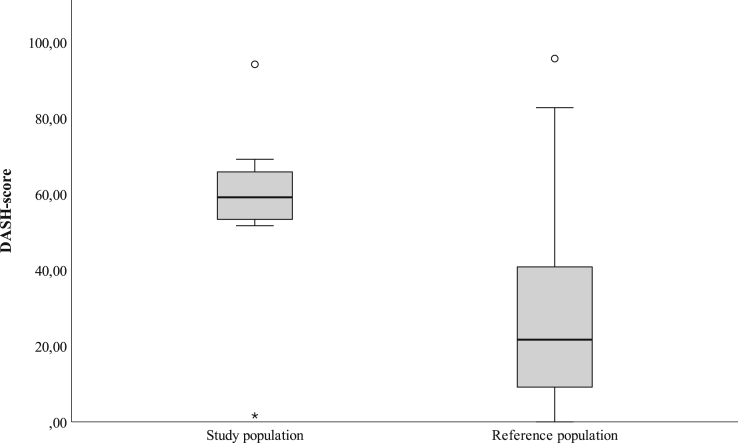
The postoperative scores from the DASH questionnaires in patients having surgery for ulnar nerve compression at a referral hospital are shown. Patients enrolled postoperatively at a specialized pain management clinic at a tertiary referral hospital, following surgery for ulnar nerve compression at the elbow, constitute the study group, while the remaining patients operated on at the same department during the same period constitute the reference group. Outliers are shown. The t test showed a statistically significant difference between the groups (*P* =.001).

Postoperative DASH scores and estimated pain VAS during postoperative activity were statistically higher for the study group than for the reference group (57 ± 22 vs. 27 ± 22, *P* =.001 and 7.3 [3.3–8.6] vs. 3.5 [1.5–5.7], *P* =.025, respectively, see Fig. 3 for DASH scores). No differences were seen in pain VAS between the groups for the other 3 subscales (ie, at rest preoperatively and postoperatively and during activity preoperatively).

The period from surgery to referral varied greatly (minimum value 7 days, maximum 794 days, median 108 days [range, 51–278 days]). Treatment provided at the pain management clinic is shown in Table 5. The most common reason for discharge from the pain management clinic was the exhaustion of treatment options (n = 10). Five patients had total pain relief or pain reduction with treatment. For another 5 patients, treatment continued through another clinic. Two patients failed to attend appointments, another 2 were discharged due to no obvious neuropathic pain component. The reason for discharge in the remaining 2 patients was unknown.

**Table 5:** Treatment Provided at Pain Management Clinic

	n (%)
Transdermal opioids	2 (9)
Transdermal capsaicin	2 (9)
Transdermal lidocaine	5 (22)
Weak opioids	1 (4)
Oral opioid combinations	5 (22)
Duloxetine	11 (48)
Non-steroid anti-inflammatory drugs	5 (22)
Tricyclic antidepressants	10 (44)
Anticonvulsants eg, gabapentin, pregabalin	10 (44)
Spinal Cord Stimulation	
Yes	1 (4)
Test	4 (17)
Transcutaneous electrical nerve stimulation (TENS)	4 (17)
Ketamine infusion	1 (4)
Series of peripheral nerve blocks	3 (13)
Conversational therapy	4 (17)
Occupational and physiotherapy	6 (26)

## Discussion

This study illustrates that postoperative pain can be a major concern for patients having surgery for ulnar nerve compression at the elbow at a tertiary referral center. We found that the study group, comprised of patients who enrolled postoperatively in treatment at a specialized pain management clinic, differed significantly in many ways from a reference group without severe postoperative pain.

The study group was significantly younger than the reference group. In other studies of surgery for ulnar nerve compression, young age has, in contrast, been associated with a more favorable surgical outcome.^33^, ^34^, ^35^ These studies do not, however, review pain as a separate outcome variable. Smoking has been associated with a worse outcome and a risk of developing chronic pain after surgical procedures in several studies,^36^ while other studies state that smoking does not affect outcome after surgery.^7^^,^^37^^,^^38^ In the present study, a high proportion of the patients were smokers. Our previous study indicated that smoking doubled the risk of complications after surgery for ulnar nerve compression, with pain after surgery being the most common complication.^9^

Higher BMI may reduce subjective well-being and increase depressive symptoms.^39^ There was a descriptive difference where patients in the study group had a higher BMI than the reference group; however, it was not statistically significant when comparing the 2 groups, (Table 1). Furthermore, the HADS scores indicated that almost half the study group, all females, suffered from clinically-relevant depression or anxiety. It is well known that pain and depression can be concurrent,^40^ which emphasizes the need to preoperatively consider mental health. Patients with mental health issues have also been shown to have a higher postoperative *Quick*DASH score and a higher rate of dissatisfaction after surgery for carpal tunnel syndrome.^41^ Unfortunately, the HADS scores could not be compared to a national representative sample due to the skewed nature of our data.

The TSK yielded high values with a degree of kinesiophobia comparable to patients with chronic back pain.^24^ A high degree of kinesiophobia has been related to a lower degree of pain-coping.^26^ The EQ-5D-index in the study group was 0.25; a value much lower than that in the general Swedish population (range, 0.74–0.89).^42^ Pain and discomfort were, as expected, the domain with the highest frequency of severe problems. The EQ-5D-VAS score was also low when compared to the general Swedish population; again, showing this condition's coexistence with low health status. Most patients were dissatisfied with almost every aspect of the LiSat-11 questionnaire, with relationships and family life being the only exceptions.

The high frequency of severe postoperative pain and low rate of pain relief observed in our study, compared to the literature, might be explained by our broad inclusion criteria or that the department is a tertiary center where severe cases are treated. More than half of the study group suffered from another ipsilateral neuropathy; a condition that is often an exclusion criterion in other studies. Another reason for this discrepancy could be the insufficient consideration of pain as a separate variable in other studies. Of the present group, 21 of 26 patients did not have a documented pain relief after treatment at the pain management clinic, indicating that such a population needed special care, a different treatment approach before surgery, or even treatment options other than surgery. The low observed rate of pain relief at the pain management clinic may not, however, show the full picture. Five patients continued treatment at another clinic, indicating that the pain management clinic had found a therapy that relieved the pain, but that it did not necessarily need to be administrated by a pain specialist. Failure to attend appointments (2 patients) might also suggest that the severity of symptoms declined.

The postoperative DASH scores differed significantly between the study and reference groups. However, there was great variation within the 2 groups, and some patients in the reference group scored higher than patients in the study group. All low postoperative DASH scores (<40), except for 1 outlier in the study group, were found in the reference group, indicating that pain greatly affected disability.

Recommendation of opioids for the treatment of neurogenic pain is very infrequent or nonexistent.^43^ The most frequently prescribed drugs at the pain management clinic were duloxetine, anticonvulsants (eg, gabapentin and pregabalin), and tricyclic antidepressants. This was in line with evidence-based recommendations for neurogenic pain.^43^^,^^44^ By only prescribing opioids when indicated, ie, not for neurogenic pain, surgeons can assist in the goal of reducing opioid misuse and abuse.^17^

There are several weaknesses in this study. The major ones include the retrospective design, the lack of preoperative and reference group SQRP-questionnaires, a low rate of questionnaire completion, and the absence of preoperative and repeated responses for questionnaires (including DASH) and pain scales. The strengths of the study include the broad inclusion criteria that are representative of clinical practice and the use of several patient-reported outcome measures. It is important to emphasize that even though the characteristics of patients with severe postoperative pain may represent predictive factors for the development of pain, no such conclusion can be drawn from this study. Further studies are needed to explore causality.

We provide a perspective that is seldom highlighted, ie, the consideration of severe postoperative pain in patients with surgery for ulnar nerve compression at the elbow. We conclude that previous pain conditions are common among patients who postoperatively enroll in treatment at a pain management clinic, and patients who enrolled are younger, more often have bilateral surgery, and have higher postoperative DASH scores than a reference group. Pain is a major concern for affected patients, and it negatively impacts disability, fear of movement, levels of anxiety and depression, overall health status, and life satisfaction. Of patients treated at the pain management clinic, 20% achieve pain relief or pain reduction, while almost 40% are discharged due to the exhaustion of treatment options. A qualitative study acquiring information on how this affects a patient’s everyday life would be helpful in further designing strategies to address this patient population. Furthermore, future studies are needed to predict the potential risk factors for the development of severe postoperative pain.
